# Prion protein—Semisynthetic prion protein (PrP) variants with posttranslational modifications

**DOI:** 10.1002/psc.3216

**Published:** 2019-11-12

**Authors:** Stefanie Hackl, Christian F.W. Becker

**Affiliations:** ^1^ University of Vienna, Faculty of Chemistry Institute of Biological Chemistry Vienna Austria

**Keywords:** glycosylphosphatidylinositol (GPI) anchor, membrane interaction, prion protein (PrP), protein semisynthesis

## Abstract

Deciphering the pathophysiologic events in prion diseases is challenging, and the role of posttranslational modifications (PTMs) such as glypidation and glycosylation remains elusive due to the lack of homogeneous protein preparations. So far, experimental studies have been limited in directly analyzing the earliest events of the conformational change of cellular prion protein (PrP^C^) into scrapie prion protein (PrP^Sc^) that further propagates PrP^C^ misfolding and aggregation at the cellular membrane, the initial site of prion infection, and PrP misfolding, by a lack of suitably modified PrP variants. PTMs of PrP, especially attachment of the glycosylphosphatidylinositol (GPI) anchor, have been shown to be crucially involved in the PrP^Sc^ formation. To this end, semisynthesis offers a unique possibility to understand PrP behavior *in* *vitro* and *in* *vivo* as it provides access to defined site‐selectively modified PrP variants. This approach relies on the production and chemoselective linkage of peptide segments, amenable to chemical modifications, with recombinantly produced protein segments. In this article, advances in understanding PrP conversion using semisynthesis as a tool to obtain homogeneous posttranslationally modified PrP will be discussed.

## Graphical abstract

The key pathophysiologic event in prion diseases is based on a conformational change of cellular (PrP^C^) into scrapie prion protein (PrP^Sc^) and is closely linked to posttranslational modifications (PTMs). Semisynthesis offers a unique opportunity to study the impact of PTMs on prion conversion, transmission, and pathogenicity, which is the major focus of this review.

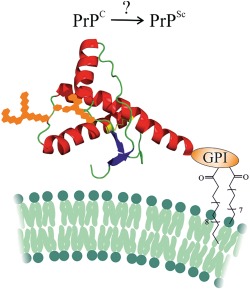

**Abbreviations**
aa: amino acid
Aβ: amyloid‐β
AD: Alzheimer disease
ADAM: a disintegrin and metalloproteinase
Adgrg6: adhesion G protein–coupled receptor G6
AFM: atomic force microscopy
AL: amyloid light chain
ALS: amyotrophic lateral sclerosis
ATPase: adenosine 5′‐triphosphatase
BSE: bovine spongiform encephalopathy
CBD: chitin‐binding domain
CCV: clathrin‐coated vesicle
CD: circular dichroism
Cdc‐42: cell division control protein 42 homolog
CDP: chronic demyelinating polyneuropathy
CFC: cell‐free conversion
CJD: Creutzfeld‐Jakob disease
CNS: central nervous system
cryo‐EM: cryo electron microscopy
CWD: chronic wasting disease
DOPC: 1,2‐dioleoyl‐*sn*‐glycero‐3‐phosphocholine
DRM: Detergent‐resistant membrane
E coli: Escherichia coli
eQuIC: enhanced quaking‐induced conversion
EPL: expressed protein ligation
ER: endoplasmic reticulum
ESR: electron spin resonance
FFI: fatal familial insomnia
FTD: frontotemporal dementia
GPI AP: glycosylphosphatidylinositol anchored protein
GSS: Gerstmann‐Sträussler‐Scheinker syndrome
Hsp: heat shock protein
KD: knockdown
K_d_: dissociation constant
LC: light chain
LRP1: low‐density lipoprotein receptor‐related protein 1
MESNA: sodium 2‐mercaptoethanesulfonate
MSA: multiple system atrophy
Mxe: mycobacterium xenopi gyrA
NCL: native chemical ligation
NMR: nuclear magnetic resonance
OR: octapeptide repeats
ORF: open reading frame
PAc: phenacyl
PD: Parkinson disease
PDB: protein data bank
PEG: polyethyleneglycol
PI‐PLC: phosphatidylinositol‐specific phospholipase C
PIRIBS: parallel in‐register intermolecular β‐sheet
PK: proteinase K
PMCA: protein misfolding cyclic amplification
POPG: 1‐palmitoyl‐2‐oleoyl‐*sn*‐glycero‐3‐phospho‐(1′‐rac‐glycerol) (sodium salt)
PPO: polyethyleneglycol polyamide oligomer
PrP: prion protein
PrP^C^: cellular prion protein
PrP^res^: (PK‐)resistant prion protein
PrP^Sc^: scrapie prion protein
PTM: posttranslational modification
PTS: protein *trans*‐splicing
QuIC: quaking‐induced conversion
RT‐QuIC: real‐time quaking‐induced conversion
ScN2a: Scrapie‐infected mouse neuroblastoma cells
SHa: Syrian hamster
SPPS: Solid phase peptide synthesis
S‐QuIC: standard quaking‐induced conversion
TEV: tobacco etch virus
ThT: thioflavin T
TSEs: transmissible spongiform encephalopathies

## 1 PRION DISEASES

Prion diseases or transmissible spongiform encephalopathies (TSEs) are incurable, neurodegenerative disorders affecting humans and animals.^1^ They include scrapie of sheep and goats, bovine spongiform encephalopathy (BSE) of cattle, chronic wasting disease (CWD) of cervids, and several human diseases such as kuru, Creutzfeld‐Jakob disease (CJD), Gerstmann‐Sträussler‐Scheinker syndrome (GSS), and fatal familial insomnia (FFI). The disease progression is accompanied by the loss of cognitive skills and neuronal dysfunction and can be of inherited sporadic or iatrogenic origin.^2^, ^3^ The central pathophysiologic event is ascribed to the conformational change of the cellular prion protein (PrP^C^) into scrapie prion protein (PrP^Sc^) that then not only propagates further PrP^C^ misfolding in neighboring cells but can also infect other organisms.^4^ Identification of the infective pathogen of prion diseases and its proof of transmissibility started in the 1950s. By ending cannibalism within the Fore tribe in Papua New Guinea, the transmission of kuru could be stopped. Experiments with transferring brain samples of these kuru victims into primates induced spongiform encephalopathies.^5^ Due to its infective property, the pathogen was first assumed to be of nucleic acid‐based, viral nature. However, the application of ultraviolet and ionizing irradiation failed to inactivate the agent, leading to the “protein‐only hypothesis” by Griffith in 1967.^6^, ^7^ Eventually in 1982 the term “prion” defining a small proteinaceous infectious particle was introduced by Prusiner during the course of discovering the prion protein (PrP).^8^, ^9^ PrP 27‐30 corresponding to the protease‐resistant core of PrP^Sc^ with an apparent molecular mass of 27 to 30 kDa was isolated by enriching fractions from Syrian hamster (SHa) brain for scrapie infectivity.^10^, ^11^, ^12^, ^13^ Successful Edman degradation paved the way for subsequent molecular cloning studies of the PrP gene.^14^, ^15^, ^16^ The linkage of PrP^Sc^ to prion diseases was recognized as an important feature of the protein, together with its role in transmission and pathogenesis of these illnesses.^17^ Thus, the main focus of elucidating prion pathogenicity is assigned to PrP. Understanding the key features in prion diseases can serve as paradigm for other neurodegenerative diseases, including amyotrophic lateral sclerosis (ALS), frontotemporal dementia (FTD), Alzheimer disease (AD), and Parkinson disease (PD), that are characterized by misfolded proteins “prionoids” sharing the aggregation properties but being not strictly infectious.^18^, ^19^, ^20^ As it happens, the latter statement might not be entirely correct. Recent prion research reported the discovery of α‐synuclein prions^21^ in multiple system atrophy (MSA) and iatrogenic AD with evidence of transmissibility of amyloid‐β (Aβ),^22^ hence highlighting the need to understand prion transmission and toxicity even more.

## 2 PROPERTIES AND STRUCTURES OF THE PrP

### 2.1 Cellular prion protein

**Figure 1:**
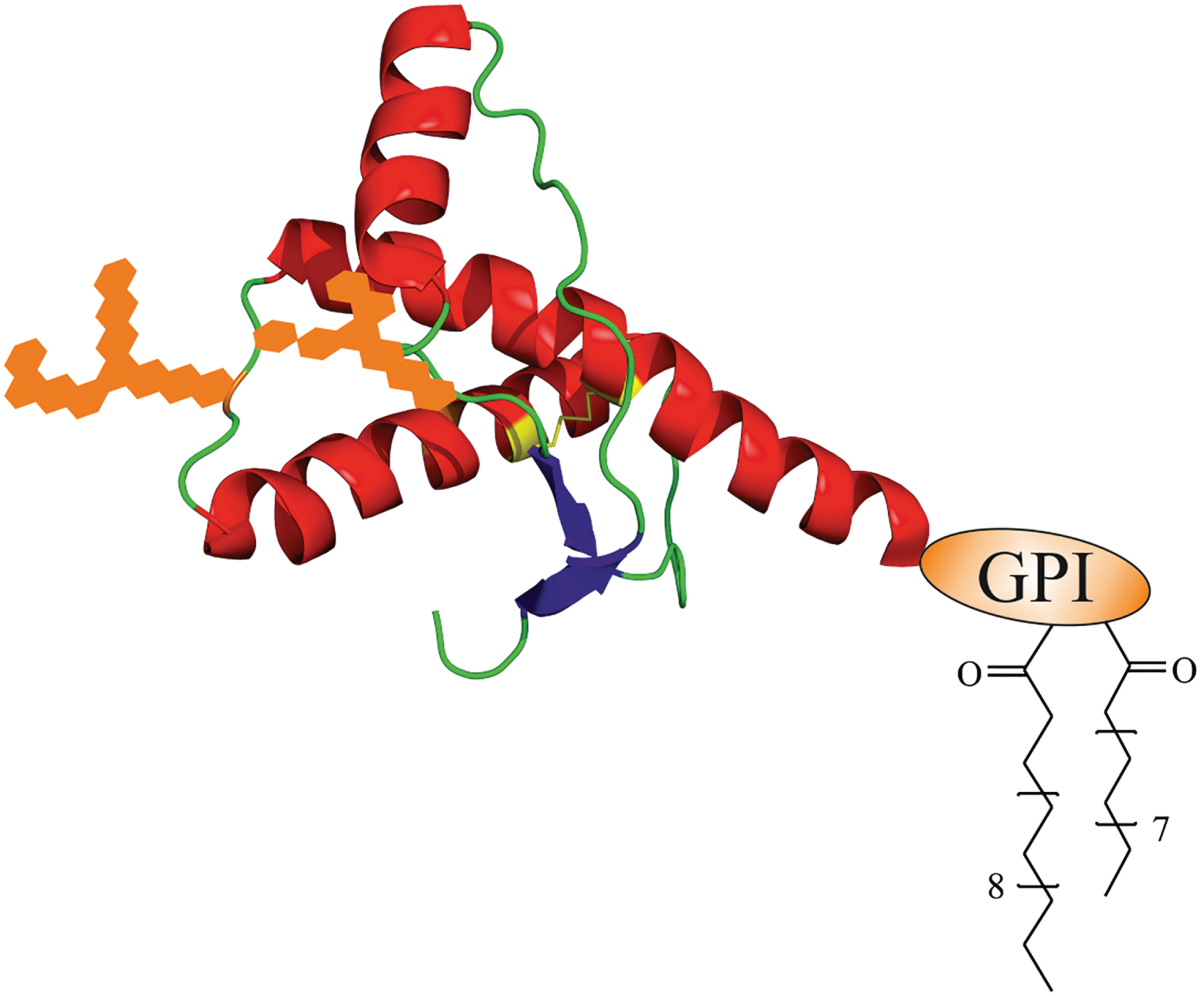
Tertiary structure of cellular prion protein (PrP^C^) with posttranslational modifications (PTMs). The structure is based on nuclear magnetic resonance (NMR) measurements of human PrP^C^ (aa 23‐230) by Zahn et al (PDB 1QLZ)^47^

High expression levels of PrP^C^ are found in the central nervous system (CNS), but it exists in other cell types and tissues, such as lymphoid organs, as well.^23^, ^24^, ^25^, ^26^ Accessing the gene‐encoding SHaPrP^C^, *Prnp*,^14^, ^27^ entailed its further identification in numerous other species and illustrated a highly conserved sequence.^28^, ^29^ The entire open reading frame (ORF) is contained within a single exon and primarily translates into a protein composed of 254 amino acids (aas).^30^, ^31^ The first 22 aas reflect an *N*‐terminal signal sequence for PrP entering the secretory pathway. Upon its cleavage, glycosylation at asparagine residues and formation of a disulfide bond occur in the endoplasmic reticulum (ER). Lastly, cleavage of the *C*‐terminal signal sequence facilitates the attachment of the glycosylphosphatidylinositol (GPI) anchor, providing mature, posttranslationally modified PrP at the outer leaflet of the cell membrane, typical for glycosylphosphatidylinositol anchored proteins (GPI APs).^32^ Interestingly, PrP can be found in three topologic forms at the ER. Apart from the fully translocated PrP, two transmembranal types occur with the *N*‐ or *C*‐terminus facing the ER lumen, denoted as ^Ntm^PrP or ^Ctm^PrP, respectively.^33^, ^34^ Normally, ^Ntm^PrP and ^Ctm^PrP only comprise a small portion of PrP^C^, whereas an excess of ^Ctm^PrP induces neurotoxicity. Neuronal cell death is caused in the absence of PrP^Sc^ formation, obviously by an aberrant metabolism of PrP^C^. PrP^C^ mislocalization represents another mechanistic possibility for prion toxicity next to the alteration of PrP^C^‐mediated signaling and PrP‐derived oligomeric species.^23^ First structural studies on PrP^C^ isolated from brains of SHas demonstrated a predominantly α‐helical content.^35^ As these measurements agreed well with subsequent spectroscopic data of recombinant PrP, accessible in larger amounts, it was considered an appropriate surrogate in biochemical experiments,^36^, ^37^, ^38^, ^39^ as well as in solving nuclear magnetic resonance (NMR) and crystal structures of PrP.^40^, ^41^, ^42^, ^43^, ^44^, ^45^, ^46^, ^47^ The PrP structure comprises an unstructured *N*‐terminal (aa 23‐120) and a globular *C*‐terminal part (aa 121‐231) (Figure 1).

**Figure 2:**
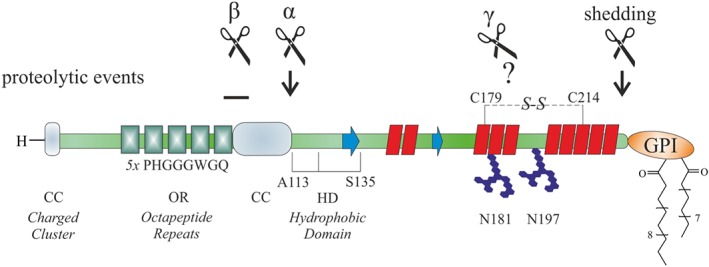
Schematic outline of the primary cellular prion protein (PrP^C^) structure. Residue numbers correspond to hPrP^C^

In more detail, the *N*‐terminal segment consists of a nonapeptide (PQGGGGWGQ) followed by four octapeptide (PHGGGWGQ) repeats (OR) with a high affinity for copper,^48^, ^49^, ^50^, ^51^ and other divalent cations,^52^ adjacent to a charged cluster (CC) or polybasic region (Figure 2). Noteworthy, the configuration of the copper binding region in hPrP (aa 23‐231) has been determined combining different experimental methods by using synthetic octapeptide and tetraoctapeptide as well as full‐length hPrP.^53^, ^54^, ^55^, ^56^ Depending on the concentration of the metal and pH, the OR region is capable to bind up to four copper ions in distinct coordination geometries.^50^, ^57^ Current estimates for dissociation constant (*K*_d_) values vary betweeen the micromolar and femtomolar range.^50^ The central hydrophobic domain (HD), comprising of aas 113 to 135, serves as a transmembrane domain^33^ and includes a palindromic region (AGAAAAGA, aa 113‐120) thought to be important in the PrP^C^‐PrP^Sc^ conversion.^58^, ^59^ Within the *C*‐terminal region, three α‐helices (aa 144‐154, 175‐193, and 200‐219), with two of them connected by a disulfide bond,^60^ and a small antiparallel β‐sheet (aa 128‐131 and 161‐164) are present. As posttranslational modifications (PTMs), a *C*‐terminal GPI anchor linked to serine 231 and two *N*‐linked glycosylation sites at asparagines 181 and 197 exist. PrP^C^ can occur in nonglycosylated, monoglycosylated, and diglycosylated forms.^61^, ^62^ Variations in glycan structures attached to PrP may be differentially distributed depending on the areas of the CNS.^63^ Molecular dynamics simulations indicate that the *N*‐linked oligosaccharides located at two helices within the structured region of PrP contribute to its stabilization in generating a negative electrostatic field covering the helical surface,^64^ thus impacting strain diversity and prion infection.^65^, ^66^, ^67^, ^68^ The *C*‐terminal GPI anchor tethers PrP to the outer leaflet of the plasma membrane.^69^

It has been postulated that mutations in the *Prnp* gene facilitate the pathogenic process by destabilizing the tertiary structure of PrP^C^. More than 30 mutations in *Prnp* could be linked to inherited prion diseases.^70^ In affecting the primary sequence of PrP, concomitant changes in its 3D structure may arise, and not cause, but influence a person's risk of developing a disease. Indeed, thermodynamic measurements of mutated PrP variants indicated destabilizing effects only for some of them.^71^ For example by comparing the wild‐type variant to the E200K mutant almost identical structures resulted, but major perturbations of the surface electrostatic potential were found. This suggests that these defects cause abnormalities in PrP interactions and should be considered as key determinants in the misfolding process.^72^

Moreover, it has been speculated that methionine oxidation in PrP^C^ plays a destabilizing role and supports spontaneous conversion into PrP^Sc^. Wolschner et al^73^ found a strong proaggregation behavior for hPrP^C^ with oxidized methionine residues and a variant with methionine replaced by hydrophilic methoxinine as a stable substitute for oxidation‐sensitive methionine. These findings suggest a pivotal role of oxidative stress in PrP conversion.

### 2.2 Scrapie prion protein

PrP^Sc^ is the toxic, misfolded isoform of PrP. It is, as PrP^C^, encoded by the *Prnp* gene and exhibits identical PTMs, but distinct structural, biochemical, and physiological features.^13^ Despite a large interest in elucidating the structure of PrP^Sc^, there are only limited data about its molecular details available.^74^ To date, obtaining a high‐resolution structure of PrP^Sc^ has been impaired by its insolubility, propensity to aggregate, and heterogeneity. Structural variations, such as differences in the glycosylation patterns, suggested to correlate with biochemical changes, including the extent of the proteinase K (PK) resistance, the electrophoretic mobility of the proteolytic fragments, and the conformational stability, depend on the distinct strains and complicate the determination of PrP^Sc^ structure.^75^ Besides, in agreement with discussions from the Prion 2018 round tables,^76^ the diversity of PrP assemblies implicates that there may be no single PrP^Sc^ structure. Data generated by biochemical and physical methods, such as spectroscopy analysis, electron microscopy, and limited proteolysis, have led to several 3D structural models. Govaerts and colleagues suggested that left‐handed β‐helices assembled into trimers, also known as the 4‐rung β‐solenoid model.^77^ Based on electron spin resonance (ESR) measurements, Cobb et al proposed a parallel in‐register intermolecular β‐sheet (PIRIBS) architecture where PrP^Sc^ consists of β‐strands and short turns and/or loops with no residual α‐helices.^78^ Still, so far, all models display discrepancies with experimental data.^79^

Notably, cryo electron microscopy (cryo‐EM) is a technique providing high‐resolution structures.^80^, ^81^ By rapidly cooling samples, proteins can be observed in their native state. In 2016, Wille and colleagues^82^ employed cryo‐EM to analyze GPI anchorless PrP 27‐30 amyloid fibrils. PrP 27‐30 was purified from brains of transgenic mice infected with prions. Further inoculation of wt mice with the purified GPI anchorless PrP 27‐30 confirmed the development of typical neurological signs of prion disease. The structure of GPI anchorless PrP 27‐30 amyloid fibrils was found to agree with a 4‐rung β‐solenoid architecture; 3D reconstruction revealed two distinct protofilaments and an average molecular height of approximately 17.7 Å. However, Collinge, Wadsworth, and coworkers^83^ studied the structural features distinguishing infectious ex vivo mammalian prions from noninfectious fibrillar assemblies generated in vitro. Applying cryo‐EM and atomic force microscopy (AFM) measurements noninfectious recombinant PrP fibrils were identified as 10‐nm‐wide single fibers with a double helical repeating substructure, agreeing with the structure described by Wille and colleagues.^82^ Prion‐infected transgenic mice replicate prions, but they mainly develop PrP amyloid plaques, which are not seen in prion‐inoculated wt mice.^84^, ^85^ Caughey and coworkers^86^, ^87^, ^88^ have described two morphologically distinct PrP fibril assemblies in prion‐infected transgenic mice. Therefore, considering the lower infecitivity titer of PrP 27‐30 in the studies of Wille and colleagues,^82^ it appears that the more abundant, single nonglycosylated PrP fibrils, corresponding essentially to recombinant PrP, has been described rather than the infectious glycosylated PrP rods. Collinge, Wadsworth, and coworkers^83^ characterized infectious PrP rods, 20 nm in width, that contained two fibers, each with a double helical repeating substructure separated by a central gap of 8 to 10 nm. The gap between the paired fibers consists of irregularly structured material compositionally distinct to the protein surface. Thus, it was proposed as a location of the *N*‐linked glycans of PrP. The structure of the infectious PrP rods differentiates them from all other protein assemblies so far studied in neurodegenerative diseases. This includes characterizations by cryo‐EM of tau filaments from AD^89^ and monoclonal immunoglobulin light chain (LC) fibrils from amyloid light‐chain (AL) amyloidosis.^90^ To date, cryo‐EM studies of tau and AL represent the only structural data of fibrils directly extracted from human tissue under pathologic conditions. For tau‐paired helical and straight filaments could be identified with cores made of two identical protofilaments that adopt a combined cross‐β/β‐helix structure. AL fibrils were found to be helical with a single protofilament showing a cross‐β architecture. It is widely accepted that during the PrP^C^‐PrP^Sc^ conversion, the β‐strand content increases vastly^91^, ^92^ and the PK resistance of the “folded core” (aa approximately 90‐231), as well.^9^, ^14^ Whereas PrP^C^ is dominated by α‐helices, monomeric, soluble, and highly susceptible to proteolytic digestion, PrP^Sc^ contains predominantly β‐sheets (>43%),^92^ aggregates into amyloid fibrils,^93^ is insoluble in detergents and partially resistant to proteolysis.^35^, ^94^ These biochemical differences between the PrP isoforms appear to be associated with the changes of the secondary structure in PrP^Sc^.

## 3 PHYSIOLOGY OF THE PrP

### 3.1 Function of PrP^C^

Although the relevance of PrP^C^ in TSEs is widely accepted, its physiological function remains enigmatic. Studies with PrP knockout mice have failed on this regard. Transgenic mice lacking PrP were found to develop normally.^95^, ^96^ A multitude of functions has been ascribed to PrP in different tissues, cells, and experimental settings, although not always without controversy or questionable reproducibility. Among others, PrP^C^ has been connected to developmental processes,^97^ cell adhesion,^98^, ^99^ neurite outgrowth, synapse formation,^100^, ^101^, ^102^, ^103^, ^104^ neuroprotection,^105^, ^106^, ^107^ and regulation of the circadian rhythm.^108^ Moreover, there is evidence for PrP contributing to myelin maintenance,^109^, ^110^, ^111^, ^112^ cellular homeostasis of divalent cations,^113^, ^114^, ^115^ and signaling events.^116^, ^117^, ^118^ A more detailed discussion can be found in reviews by the group of Aguzzi.^109^, ^119^ Recently, other functions have been attributed to PrP^C^, that is acting as a receptor for the aggregated proteins Aβ oligomers^120^, ^121^, ^122^ and α‐synuclein.^123^ By mediating the uptake of Aβ and α‐synuclein, PrP^Sc^ is unable to replicate in their presence.

Already 10 years ago, it was suspected that reports on the function of PrP represent just specific aspects of a more complex physiological role of PrP^C^.^23^ Causes for the functional diversity of PrP^C^ might not only be its alternating transient binding partners in different cellular locations but also its proteolytic processing.^124^, ^125^ For once, PrP fragmentation may inhibit association with some binding partners while possibly allowing new interactions with others. Then again, the cleaved products may act as soluble ligands facilitating protein interactions over large distances. These findings contribute to the biological complexity of the physiological function of PrP. In fact, four different but highly conserved cleavage events have been significantly characterized (Figure 2).^126^, ^127^, ^128^ During transport to the cell surface, α‐cleavage results in a soluble flexible *N1*‐ and a globular membrane‐bound *C1* part. Myelin maintenance has been initially linked to this *C1* part derived from α‐cleavage. Mice expressing PrP mutants not able to undergo α‐cleavage suffered from chronic demyelinating polyneuropathy (CDP).^110^, ^111^, ^129^ Interestingly, recent work found a specific ligand role of the flexible *N1* part towards the G‐protein coupled receptor Adgrg6, promoting myelin homeostasis.^112^ During shedding, PrP^C^ is released from the plasma membrane by a disintegrin and metalloproteinase (ADAM) enzyme, namely, ADAM10, in a glycosylated form without the GPI anchor and designated as “shed PrP.”^130^ Although definite functions of “shed PrP” are not known to date, the shedding process regulates the membrane levels of PrP^C^ and thus its functions at the cell surface. Similar to α‐synuclein,^123^ recent work by Jarosz‐Griffiths et al^131^ found that the toxicity and cellular binding of Aβ oligomers can be reduced by shedding of PrP^C^, thereby pointing towards a contribution as a receptor in AD. Moreover, PrP^C^ is expressed in immune cells as well, particularly on mast cells.^132^ Upon activation of these cells, the PrP^C^ shedding process is enhanced, proposing PrP involvement in the inflammatory mast cell response. Under pathological conditions and in response to oxidative stress, incidences of β‐cleavage occurring around aa position 90 are increased.^133^, ^134^ Lastly, γ‐cleavages restricted to unglycosylated PrP generate a large soluble *N3* and a short *C3* part by taking place in a region between aas 170 and 200. While prevalence and relevance of this cleavage requires further investigation, increased amounts of *C3* in CJD brain samples suggest a pathophysiological role.^135^

Despite multiple evidence of PrP in physiological processes, the functional diversity based on its manifold binding partners and proteolytic fragments complicate an exact definition of its physiological function. Yet successful elucidation of pathways and roles of PrP could help to understand its linkage to toxicity in prion diseases and to other neurodegenerative diseases.^136^

### 3.2 Trafficking of PrP^C^

As the PrP function is closely intertwined with the cellular compartments where the protein is located, having a closer look at trafficking may assist in elucidating its involvement in pathological and physiological processes. PrP^C^ is tethered via its GPI anchor to the outer leaflet of the plasma membrane.^69^ In 1993, data by Shyng et al^137^ revealed constitutive cycling of PrP^C^ between the cell surface and endocytic compartments on varying times scales dependent on the cell line, as demonstrated in later work.^138^ From the cellular membrane, PrP^C^ can enter the cell *via* multiple pathways, mediated mainly by the unstructured *N*‐terminal domain.^139^, ^140^ Evidence for a cooperation between clathrin^138^, ^141^, ^142^ and rafts^143^, ^144^, ^145^ in the internalization of PrP^C^ was found.^146^ Clathrin is a large, oligomeric protein assembling into lattice structures on the inner surface of the plasma membrane. Thereby, it causes the membrane to invaginate and pinch off to form clathrin‐coated vesicles (CCVs), which can then fuse with other intracellular organelles.^147^ Although a clathrin‐dependent internalization might appear unusual since PrP lacks a cytoplasmic domain necessary for the direct interaction with clathrin and the adaptor protein, GPI APs can indeed enter the clathrin‐dependent pathway upon interaction with transmembrane proteins possessing a clathrin‐coated pit internalization signal.^144^ Moreover, the endocytosis of PrP^C^ was found to be associated with the low‐density lipoprotein receptor‐related protein 1 (LRP1)^142^, ^148^ that belongs to a receptor family of cell‐surface transmembrane proteins capable of binding a variety of ligands and internalizing via clathrin‐coated pits.^149^, ^150^ As a nonclassical clathrin‐independent pathway, the raft‐dependent internalization route distinguishes caveolae‐dependent and caveolae‐independent endocytosis.^151^ Caveolae are membrane invaginations, originating from the oligomerization of caveolins, their integral coat proteins, and are considered to be specialized raft domains.^152^, ^153^ Due to the presence of PrP^C^ in caveolae‐like domains^154^, ^155^ and its colocalization with caveolin‐1 (cav‐1),^143^, ^156^ the involvement of caveolae in PrP^C^ endocytosis had been suggested earlier. To this end, Sarnataro et al^157^ could provide evidence that the raft‐mediated pathway is not affected by caveolin expression. Still, PrP^C^ internalization was found to be impacted by cholesterol depletion and activation of the cell division control protein 42 homolog (Cdc‐42), a member of the Rho family of GTPases being specifically involved in clathrin‐independent endocytosis of GPI APs.^158^ Additionally Sarnataro et al^157^ reported that in coimmunoprecipitation studies of clathrin and PrP^C^, the latter remained associated with detergent‐insoluble microdomains. This fact supports a cooperation between rafts and clathrin in the internalization process. PrP^C^ susceptibility to various endocytic pathways could also be the basis for its neuroprotective and neurodegenerative functions.

## 4 MECHANISM OF PrP^C^‐PrP^Sc^ CONVERSION

**Figure 3:**
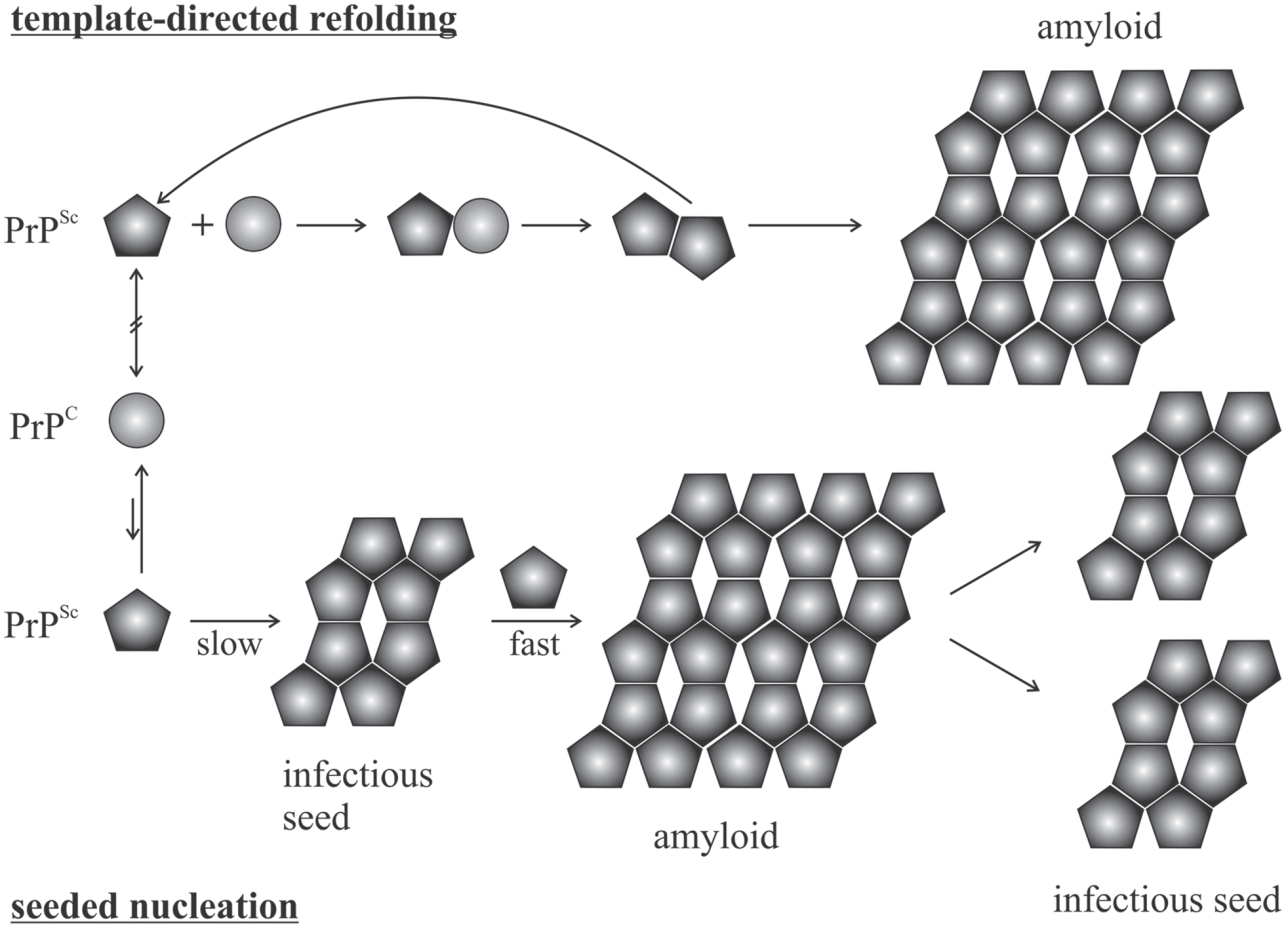
Models for the conversion of cellular prion protein (PrP^C^) into scrapie prion protein (PrP^Sc^). The model for template‐directed refolding (top) and seeded nucleation (bottom) are depicted. The figure was modified from Aguzzi and Calella^23^

To date, despite considerable knowledge about the characteristics of the infective prion pathogen, its mechanism of replication and the molecular pathways leading to neurodegeneration are largely unknown. There is evidence from *in* *vitro* and transgenic mouse studies that the conversion to PrP^Sc^ implicates PrP^C^‐PrP^Sc^ interactions.^84^, ^159^, ^160^, ^161^, ^162^, ^163^ The rate of PrP^Sc^ formation and disease progression appears to be directly proportional to the level of PrP^C^ expression, indicated by PrP knockout mice not propagating scrapie infectivity and transgenic mice heterozygous for a disrupted PrP gene requiring prolonged incubation times upon prion inoculation.^164^, ^165^, ^166^ In agreement with the “protein‐only hypothesis,” these findings have raised two models explaining prion replication (Figure 3). The template‐directed refolding model by Prusiner^167^ proposes that a high‐energy barrier prevents the spontaneous PrP^C^‐PrP^Sc^ conversion. Upon interaction, monomeric PrP^Sc^ induces PrP^C^ to convert into PrP^Sc^. However, until now, there is no experimental evidence for the existence of a stable PrP^Sc^ monomer.^168^ PrP^Sc^ seeds in this prion propagation process are not considered essential. Alternatively, in the more accredited seeded nucleation model by Jarrett and Lansbury,^169^ a reversible thermodynamic equilibrium between PrP^C^ and PrP^Sc^ is postulated. In the presence of stable oligomeric PrP^Sc^ aggregates, the conversion from PrP^C^ to PrP^Sc^ is favored, thus making PrP^Sc^ aggregates (seeds) inevitable for prion spread. Fragmentation of PrP^Sc^ aggregates increases the number of nuclei capable of recruiting further PrP^Sc^. In fact, these soluble oligomers produced during the PrP amyloid aggregation have emerged as the primary neurotoxic species, supporting the seeded nucleation model.^170^, ^171^, ^172^

Ultimately, evidence for a direct PrP^C^‐PrP^Sc^ interaction in the conversion to PrP^Sc^ came from *in* *vitro* systems. Pioneering studies from Caughey and colleagues^173^ succeeded within a cell‐free conversion (CFC) assay in the generation of protease‐resistant (res), radioactive PrP^res^ from mixed PrP^C^ substrate and an excess of unlabeled PrP^Sc^. This in vitro PrP^res^ propagation recapitulates the species and strain specificity of prion transmission *in* *vivo*.^173^, ^174^ Mechanistically, it has identified structural factors underlying the species barrier and optimal conditions for the PrP^res^ formation.^66^, ^175^, ^176^ The ability to generate PrP^res^ not only from purified but also recombinant protein^177^ provides a unique opportunity to study prion propagation. CFC assays can be used as screening experiments as they have the potential to identify compounds directly inhibiting the PrP^C^‐PrP^Sc^ interaction or its subsequent conversion.^178^, ^179^ Still, the proportionally large amount of PrP^Sc^ seeds required to drive the CFC assay (PrP^Sc^:PrP^C^ = 50:1) has prevented it from generating de novo infectivity.^180^

A more efficient method for mimicking the autocatalytic replication of PrP^Sc^ was provided by Soto and colleagues^38^ in affording a larger than 10‐fold increase in PrP^res^ with the usage of a 1:100 ratio of PrP^Sc^ to PrP^C^. By subjecting scrapie‐infected and normal brain homogenate to the so‐called protein misfolding cyclic amplification (PMCA) procedure, PrP^res^ is amplified in cycles of sonication and incubation. Successive rounds of PMCAs and fragmentation of PrP^Sc^ rise the available amounts of replication‐competent species.^181^, ^182^, ^183^ Thus, with automation, this assay offers a promising diagnostic tool in pre‐symptomatic blood screening,^184^, ^185^ and eventually, it has facilitated the detection of de novo infectivity in hamsters.^38^ However, the levels of infectivity still remain lower than with a similar quantity of brain‐derived PrP^Sc^, and the usage of complex brain homogenate itself represents an obstacle in thoroughly elucidating the conversion and association of infectivity with PrP^res^.^186^ Besides, the distinct efficiency differences between the CFC and PMCA assays applying purified and crude brain‐derived PrP^Sc^ proposed that cellular accessory factors are involved in the generation of PrP^res^. In fact, polyanionic molecules were identified as factors present in the brain homogenate that contribute to the conversion efficiency.^187^ Ongoing development of PMCA assays aiming to detect and early diagnose TSEs has led to the quaking‐induced conversion (QuIC) method.^188^ Sonication is replaced by reproducible and easier controllable shaking during the prion amplification process, which enables the application of standardized protocols. This accomplishment is reflected by the multiple variations currently available, such as standard (S‐QuIC), real‐time (RT‐QuIC), and enhanced QuIC (eQuIC).^189^, ^190^, ^191^, ^192^

Apart from the autocatalytic propagation of PrP^Sc^, another crucial hallmark of the PrP^C^‐PrP^Sc^ conversion is the *de novo* generation of infectivity. However, when inoculated into animals, PrP fibrillar assemblies can range from being biologically inert to fully infectious, pathogenic, and transmissible in subsequent passages.^37^, ^76^, ^193^, ^194^, ^195^ Legname and coworkers^196^ inoculated transgenic mice expressing truncated PrP^C^ (aa 89‐231) with amyloid fibrils formed from recombinant PrP (aa 89‐230). The outcomes were low infectious titers and the affection of only that single line of transgenic mice Tg9949, probably due to the high expression and truncation of the transgene sequence enhancing the susceptibility to prion infection within the mice.^75^ Hence, according to Supattapone,^197^ these highly concentrated samples of PrP amyloid fibrils are not suitable in mimicking the infectious properties of PrP^Sc^. In contrast, Wang et al^39^ succeeded in the formation of infectious *de novo* recombinant PrP amyloid fibrils associated with neurological signs in wild‐type mice after approximately 130 days. Here, PrP^res^ was formed in PMCA assays in the presence of negatively charged lipids, namely, 1‐palmitoyl‐2‐oleoyl‐*sn*‐glycero‐3‐phospho‐(1′‐rac‐glycerol) (sodium salt) (POPG). In their earlier work,^198^ they had shown that POPG promotes the conversion to PrP^res^ under physiological conditions. In further studies, Wang et al^199^ could attribute a crucial role in the PrP‐lipid interaction to the highly conserved middle region of PrP that induced conformational change.

### 4.1 PrP^C^‐PrP^Sc^ conversion in cells

The findings regarding PrP^res^ formation in the presence of POPG^39^ support the possibility of the plasma membrane being the cellular localization of PrP^Sc^ formation as a posttranslational event. At this position, contact between endogenous PrP^C^ and exogenous PrP^Sc^ can easily occur.^200^, ^201^, ^202^, ^203^, ^204^ This is supported by the finding that by releasing PrP^C^ from the cell surface or interrupting its transport to the plasma membrane prevents the formation of PrP^Sc^.^205^, ^206^, ^207^ More precisely, both PrP isoforms were found to be associated with rafts.^208^, ^209^, ^210^, ^211^ These are defined as highly dynamic microdomains wherein specific lipids stabilize larger lipid platforms and compartmentalize cellular processes at the membrane.^212^ Impairing the integrity of the cholesterol‐enriched rafts associated with PrP by lowering the intracellular levels of cholesterol reduced the formation of PrP^Sc^ in infected cells.^213^ Moreover, PrP^C^‐ and PrP^Sc^‐associated rafts were found to have distinct characteristics, as they can be separated from each other by solubilization and flotation on density gradients.^208^ According to Campana et al,^200^ this proposes that either the types of raft or the membrane association of each isoform has different characteristics. However, Baron et al^209^ illustrated that the PrP^Sc^‐PrP^C^ conversion only takes place in the presence of fused PrP^Sc^‐ and PrP^C^‐containing membranes, suggesting that the two PrP isoforms need to be inserted into contiguous membranes. Alternatively, rafts were proposed to stabilize PrP in its conformation via a direct interaction with cholesterol. Thus, changes in the local lipid environment can mediate PrP conformation.^214^ Studies on model lipid bilayers regarding the impact of the PrP‐lipid interaction on structure and affinity of PrP support the idea that predominantly α‐helical PrP^C^ is stabilized upon binding to raft membranes, whereas binding to negatively charged lipid (nonraft) membranes leads to an increased β‐sheet content.^215^ Interestingly, the PrP‐raft association is mediated by the GPI anchor^213^, ^216^ and the *N*‐terminal region of PrP.^217^ Unlike for a typical GPI‐anchored protein, for PrP, this raft association occurs already earlier in the secretory pathway and appears to be involved in the maturation and folding process of PrP^C^.^218^, ^219^ Alternatively to the plasma membrane, the formation of PrP^Sc^ was suggested to involve additional cellular places. Immediately after PrP internalization, the PrP^C^‐PrP^Sc^ conversion may occur in the endolysosomal compartment,^205^ in the Golgi apparatus and/or the ER following retrograde transport.^220^, ^221^ In infected cells, stimulation of retrograde transport towards the ER leads to an increase in PrP^Sc^ formation from PrP^C^ precursor,^222^ suggesting that the ER may represent an amplification compartment for PrP^Sc^.^223^ Participation of the endocytic pathway is indicated by PrP^Sc^ accumulation in the late endosomes.^205^, ^211^, ^224^, ^225^ Still, as demonstrated by Goold et al,^201^ the plasma membrane is the initial site of prion conversion and consequently of most interest in studying the earliest events in prion infection and PrP misfolding.

### 4.2 Impact of GPI anchor on PrP^C^‐PrP^Sc^ conversion

Typically, PrP is attached to membranes by its GPI anchor (Figure 6A).^69^ A better understanding of the interplay between membranes, GPI‐anchored PrP, and PrP^C^‐PrP^Sc^ conversion is provided by work from Baron and Caughey.^209^, ^210^ First, they studied the conditions necessary for PrP^res^ formation of PrP associated with detergent‐resistant membranes (DRMs).^209^ Based on that, in CFC assays, Baron and Caughey^210^ investigated the impact of GPI‐anchoring of PrP associated with model membranes on PrP^res^ formation. PrP was isolated by immunoprecipitation from mammalian cell lines expressing GPI‐anchored and anchorless PrP, respectively.^173^, ^226^, ^227^ GPI‐anchored PrP bound to liposomes could not be converted to PrP^res^ upon exposure to exogenous PrP^res^ in microsomes until phosphatidylinositol‐specific phospholipase C (PI‐PLC) was added or the combined membrane fractions were treated with a membrane‐fusing agent. These findings indicate for the initiation and propagation of PrP^Sc^ that at the membrane surface, an insertion of PrP^Sc^ into the host cell membrane is necessary for the conversion. Whereas if the conversion occurred extracellularly, PrP^C^ needed to be released from the cell membrane. In contrast, anchorless PrP bound to liposomes was converted to PrP^res^ without any treatments necessary. Hence, contradictory to PrP conversion occurring at the cellular membrane,^205^, ^206^, ^207^ only the membrane‐associated form containing PrP attached to a GPI anchor could resist the conversion induced by exogenous PrP^res^. Moreover, Chesebro et al^84^ found that anchorless PrP results in infectious amyloid disease but without typical clinical TSE. Scrapie infection of transgenic mice lacking GPI‐anchored PrP^C^ leads to a formation of amyloid plaques in contrast to nonamyloid deposits, typically observed in wild‐type mice. Although neuropathological lesions were induced, clinical manifestations were minimal. Surprisingly, the combined expression of anchorless and wild‐type PrP accelerated the onset of clinical disease. This suggests that GPI‐anchored PrP may be critically involved in the pathogenesis of prion diseases.^228^ Overall, the findings mentioned above indicate a major contribution of the GPI anchor in the toxicity of the PrP^C^‐PrP^Sc^ conversion.

## 5 TOWARDS THE ELUCIDATION OF PrP CONVERSION

Recombinant PrP is an appropriate surrogate for PrP^C^, as determined by spectroscopic measurements, including circular dichroism (CD), that eventually facilitated solving the NMR and crystal structures of PrP.^40^, ^41^, ^42^, ^43^, ^44^, ^45^, ^46^, ^47^ However, it can be a suitable representative only under certain conditions, including thioflavin T (ThT) fluorescence‐based following of the aggregation process. Even though with this method, insights into the characteristics and kinetics of in vitro fibril formation have been gained,^232^ just recently, the molecular basis of PrP replication was established in detail by applying a single‐molecule fluorescence methodology to characterize individual aggregates. With total internal reflection fluorescence (TIRF) microscopy, Klenerman and colleagues^233^, ^234^ studied fibril fragmentation and elongation of individual murine PrP aggregates from seeded aggregation *in* *vitro*. PK‐resistant PrP fibrils elongated until length‐dependent fragmentation resulted in PK‐sensitive fragments. This method allowed direct observation of heterogeneous, transient, metastable oligomers during aggregation, found to be the most infectious PrP particles.^235^ Additionally, a spreading model for aggregate propagation through the brain could be predicted, and a framework was established to start determining the main factors that control the rate of prion spreading in animals. In 2011, Goold et al^201^ analyzed a PrP knockdown (KD) neuroblastoma cell line expressing epitope‐tagged PrP^C^ upon infection with exogenous PrP^Sc^. After facing the limitation of immunological differentiation between PrP^Sc^ and PrP^C^ expressed on the recipient cell from cell lines susceptible to prion infection, epitope‐tagged PrP^C^ appeared an elegant solution. However, several previous attempts had failed in generating PrP molecules capable of prion conversion, probably due to the sequence sensitivity in this process, particularly in certain key regions of the PrP molecule.^236^, ^237^, ^238^, ^239^ Eventually, out of eight different constructs, Goold et al^201^ succeeded with a PrP‐224AlaMYC construct, in which the tag is inserted within the *C*‐terminal domain. A detailed analysis of the cells shortly after prion exposure demonstrated that PrP^Sc^ is formed on the plasma membrane. Furthermore, PI‐PLC treatment effectively removed PrP^C^ from the plasma membrane of PrP‐224AlaMYC cells and reduced the generation of PrP^Sc^. However, immunostaining is only feasible on fixed cells and impedes dynamic studies revealing molecular details involved in the PrP conversion and propagation processes. ThT‐based detection of preformed PrP amyloid fibrils applied in a cellular environment cannot exclude ThT binding to other structures unrelated to amyloid and guarantee binding to all aggregates, as the binding mechanism is not fully understood yet.

To this end, labeling of PrP with an organic fluorophore is required for dynamic studies in cells. Recombinant PrP differs compared with PrP^C^ in a complete lack of PTMs causing distinct infectivity and membrane interaction characteristics. These properties can be mainly ascribed to the GPI anchor, tethering PrP to the cellular membrane, as demonstrated in various studies.^84^, ^209^, ^210^, ^231^, ^240^, ^241^, ^242^ Advances in semisynthetic strategies based on solid‐phase peptide synthesis (SPPS),^243^ protein engineering, native chemical ligation (NCL),^244^ and expressed protein ligation (EPL)^245^, ^246^ have facilitated access to homogeneous membrane‐anchored labeled PrP variants that allow to directly observe the biophysical properties of PrP upon interaction with the cellular membrane.

## 6 SEMISYNTHESIS STRATEGIES FOR PrP

**Figure 4:**
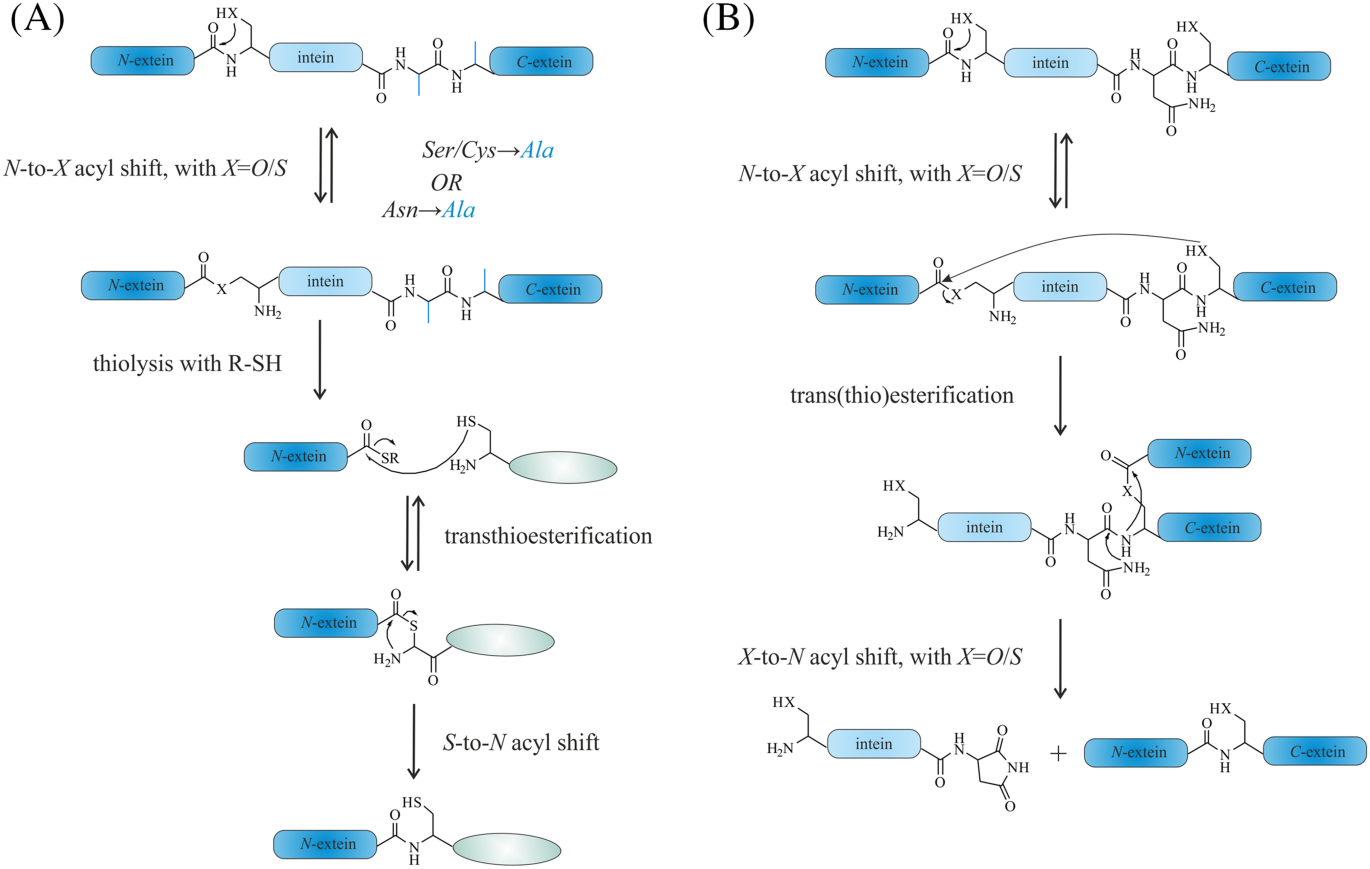
Mechanism of the expressed protein ligation (EPL) reaction (**A**) and protein *trans*‐splicing (PTS) (**B**)

**Figure 5:**
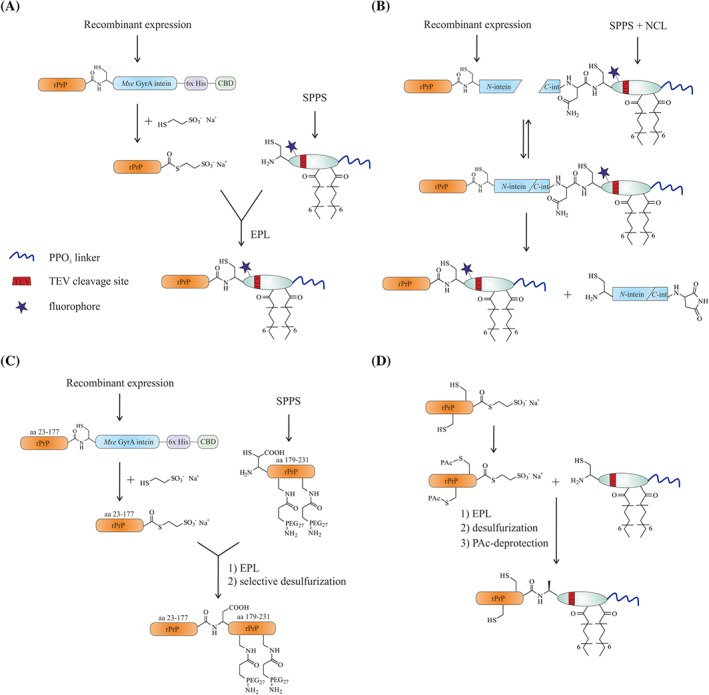
Semisynthetic strategies for prion protein (PrP) variants, developed in the Becker laboratory.^228^, ^255^, ^256^ *Via* an expressed protein ligation (EPL)‐ (**A**) and a protein *trans*‐splicing (PTS)‐based (**B**) approach, PrP variants equipped with a glycosylphosphatidylinositol (GPI) anchor mimic can be obtained. With the strategy displayed in **C**, PrP variants modified with monodisperse PEG chains as mimics of *N*‐glycans can be accessed. Selective desulfurization of the introduced cysteine following EPL (**A**) is depicted in **D**

To date, the majority of studies on the function and structure of PrP have been carried out with recombinant protein lacking all PTMs, including the GPI anchor, or with heterogeneous protein preparations isolated from mammalian cell lines.^247^, ^248^, ^249^ Still, there have been attempts towards generating defined membrane‐anchored PrP. Glockshuber^250^ and Pinheiro^251^ with colleagues applied similar strategies, in which thiol‐reactive lipids were attached to the *C*‐terminus of recombinant PrP carrying a cysteine. However, further application of these PrP constructs in cell‐based assays may be impeded by potential side reactions with thiol‐containing compounds or internal cysteines of PrP, as the lipids were attached via disulfide bonds. Different strategies were utilized by Baskakov,^252^ Baldwin,^253^ and Moroder^254^ with coworkers: Baskakov and colleagues^252^ applied maleimide chemistry to introduce a myristoyl chain at the *C*‐terminus of genetically engineered PrP(S230C). This modification did not alter the structure of the protein. Interestingly, an increasing affinity of PrP for the cell membrane and a decreased extent of fibrillization was found. Baldwin^253^ and coworkers chemically synthesized several PrP segments, including a 106 residue “mini‐prion” (PrP106) by connecting PrP (aa 90‐141) to PrP (aa 178‐231) *via* a native peptide bond using NCL,^244^ a selective reaction that links an unprotected peptide containing a *C*‐terminal α‐thioester to another peptide with an *N*‐terminal cysteine. A membrane anchor made of a lipophilic myristoyl chain was introduced at the *C*‐terminus of shorter PrP peptides *via* an orthogonally side‐chain–protected lysine. Immunofluorescence analysis indicated that only myristylated PrP peptides could be targeted to the cell surface. The group of Moroder applied click and ligation chemistry to obtain lipidated peptides corresponding to the *C*‐terminal PrP segment (aa 214‐231).^254^ Confocal images of HeLa cells revealed a direct transfer of fluorescently labeled lipopeptides to the cellular membrane. Thus, lipopeptides can be used as mimics of the GPI anchor's ability to attach PrP to the cell membrane. A similar strategy with regard to using a lipidated peptide as a GPI‐mimicking membrane anchor was pursued by Becker et al, when starting to develop semisynthetic strategies, based on EPL and protein *trans*‐splicing (PTS) (Figure 4), to access to different posttranslationally modified PrP variants (Figure 5).^228^, ^241^, ^255^, ^256^, ^257^, ^258^, ^259^, ^260^, ^261^

In the EPL^245^, ^246^ reaction, a protein thioester, obtained by cleaving a fusion protein consisting of the protein of interest (POI) and an intein, is linked to a chemically synthesized peptide^243^ containing an *N*‐terminal cysteine in a reaction similar to NCL.^244^ An initial transesterifaction leads to formation of a thioester linking the recombinant and synthetic PrP segments, and a subsequent irreversible *S* → *N* acyl shift establishes the amide bond at the ligation site (Figure 4A). The recombinant protein α‐thioesters can be accessed using engineered inteins. Inteins are self‐processing protein segments, which mediate protein splicing.^245^, ^262^ In the course of this intramolecular process, the intein excises itself and joins the *C*‐ and *N*‐terminal flanking protein segments (*C*‐ and *N*‐extein). In more detail, a nucleophilic side chain, namely, a hydroxy or thiol group for serine and threonine or cysteine residues, accomplishes an *N* → *O* or *N* → *S* acyl shift. Then, in a *trans*‐(thio)esterification, the *N*‐extein gets attached to a conserved *N*‐terminal serine or cysteine of the *C*‐extein. The instable branched intermediate is resolved via an intramolecular rearrangement involving a conserved asparagine residue of the intein producing an intein with a *C*‐terminal succinimide, and an *O* → *N* or *S* → *N* acyl shift resulting in ligated exteins with a native bond at the ligation site.^262^, ^263^ Mutations of the *C*‐terminal asparagine of the intein and the *N*‐terminal cysteine, threonine, or serine residue of the *C*‐extein to alanine block the splicing process and only allow the initial *N* → *S* acyl shift, which enables the generation of a protein α‐thioester by addition of an excess of a thiol, such as sodium 2‐mercaptoethanesulfonate (MESNA), to trap the protein thioester.^264^ PTS is a process that relies on the assembly of two divided segments of inteins, so‐called split inteins, to form a functional intein. Upon assembly of the split inteins, PTS occurs and links the *N*‐ and *C*‐exteins in a similar sequence of events as described above (Figure 4B).^265^

**Figure 6:**
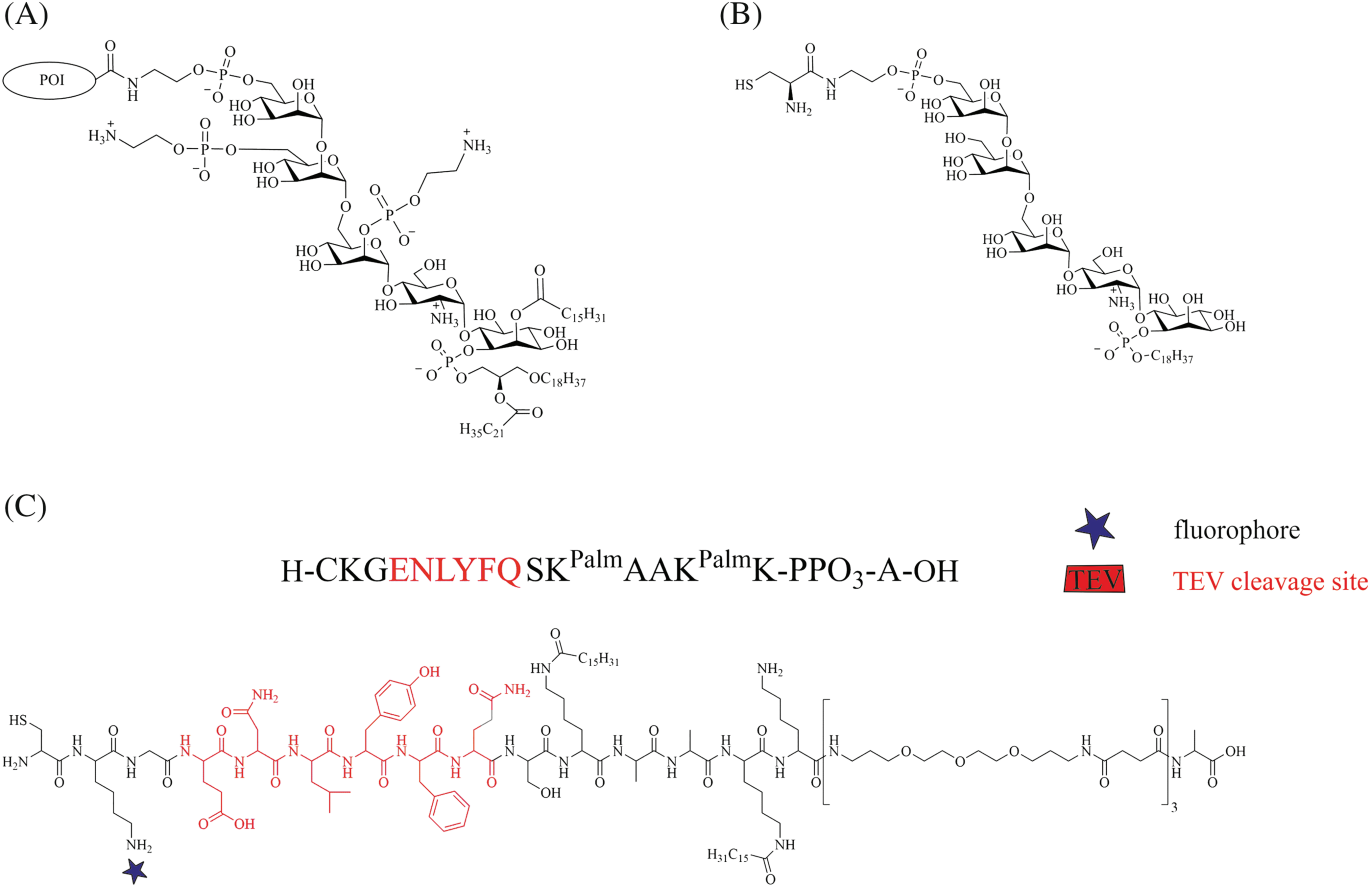
*Structures of a native and artificial glycosylphosphatidylinositol (GPI) anchor together with a GPI anchor mimic. **A** shows a common structure of a native GPI anchor from human erythrocyte acetylcholinesterase.* 226 ***B** is a chemically synthesized GPI from Silva et al* 227 *with a cysteine residue for* native chemical ligation (*NCL) reactions. **C** shows the structure of the GPI anchor‐mimicking peptide used for the semisynthesis of lipidated PrP constructs* 228

The generation and biophysical characterization of PrP constructs containing a GPI anchor mimic started more than 10 years ago in the Becker laboratory with work described in Olschewski et al.^228^ Two strategies based on the EPL approach provided PrP^Palm^, an *N*‐terminally truncated PrP variant (T_PrP [aas 90‐231]) that is missing the unfolded *N*‐terminal domain (aa 23‐89) and modified at the *C*‐terminus with chemically synthesized membrane anchor peptides (Figure 5). At that time, the protease‐resistant PrP fragment comprising residues aa 90‐231 had been considered as the structure crucially involved in TSEs. The GPI anchor mimics (Figure 6C) feature two palmitoyl modifications (Palm) that induce a high affinity towards DOPC liposomes and locate PrP in its native conformation to the detergent‐resistant domains (DRMs) of cell membranes.^266^ A tobacco etch virus (TEV) protease recognition site (ENLYFQ) facilitates controlled release of PrP from the membrane, a polyethyleneglycol (PEG) polyamide oligomer (PPO) functions as solubilization tag to handle the palmitoylated peptides in aqueous buffers,^267^ and a fluorescent dye can be incorporated for tracking of the semisynthetic PrP *in* *vitro* and *in* *vivo* (Figure 6C).

One of the initial EPL‐based strategies relies on the expression of PrP in fusion with the *Mxe* GyrA intein and a combination of two affinity tags, namely, a His tag and a chitin‐binding domain (CBD) in *Escherichia coli* (*E coli*). Cleavage of this construct is achieved with an excess of thiol to generate PrP with a *C*‐terminal thioester. This PrP‐thioester is incubated with the GPI anchor‐mimicking peptides and gives the *C*‐terminally modified PrP (denoted as PrP^Palm^, Figure 5A). A second strategy is based on PTS by expressing PrP fused to the *N*‐terminal segment of the DnaE split intein (DnaE^N^) and chemical synthesis of its *C*‐terminal segment (DnaE^C^, 36 aa) linked to the GPI anchor‐mimicking peptides by a prior NCL reaction.^268^ Both DnaE segments spontaneously associate when folded and form a functional intein, which excises itself to give the desired modified PrP with its *C*‐terminal membrane anchor (Figure 5B). Aggregation assays based on PK resistance and ThT binding^269^ revealed an extended lag time for vesicle‐attached PrP^Palm^ with respect to conversion into PrP^res^ and fibril formation than for PrP in control experiments. Further, binding to zwitterionic DOPC liposomes indicated a very strong membrane interaction for PrP^Palm^ in contrast to PrP. Transfer of PrP^Palm^ onto neuronal cells gave rise to similar patterns observed for native PrP^C^ by immunostaining. Together with extraction experiments of the cell membrane, this provided proof that soluble PrP^Palm^ is attached to detergent‐resistant domains (DRMs) similar to wild‐type PrP^C^ with a native GPI anchor.

Next, Becker et al^257^ developed a synthetic strategy for the preparation of PrP with a native, homogeneous GPI anchor that can also be applied for other GPI‐anchored proteins. A challenge lies here in the chemical diversity of GPI anchors on the same protein. Different glycoforms of native PrP GPI anchors have been reported with the exact linkage positions and anomeric configuration of the oligosaccharide branches not defined. At the same time, details about the lipids attached to these GPI anchors are not fully clear (Figure 6A).^270^ In view of this structural uncertainty, a core GPI pseudopentasaccharide, containing three mannose (Man), a glucoseamine (GlcN), and an inositol (Ino) glycan connected in an α‐Man‐(1 → 2)‐α‐Man‐(1 → 6)‐α‐Man‐(1 → 4)‐α‐GlcN‐(1 → 6)‐*myo*‐Ino way, was selected. The incorporation of a cysteine residue on the 2‐aminoethyl phosphate moiety of the GPI backbone prior to global deprotection provided a synthetic, cysteine‐tagged GPI anchor suitable for NCL reactions (Figure 6B). In a following EPL reaction, PrP with a *C*‐terminal thioester was linked to this synthetic GPI anchor. Analysis of the secondary structure of PrP attached to the synthetic GPI revealed that the CD curves are indistinguishable from the spectra of PrP and comparable with the spectra of PrP^C^. Moreover, the CD spectra were found to agree with the spectra of PrP^Palm^. This observation confirms the successful application of the GPI anchor‐mimicking peptides (Figure 6C) as an alternative to circumvent the elaborate synthesis of a GPI anchor (Figure 6B). Even though the synthesis of the GPI anchor succeeded, it remains a challenge to provide sufficient amounts for subsequent experiments and extension to other proteins. Isolating mostly homogeneous, cysteine‐carrying GPI anchors from natural sources could help to avoid this problem, and first steps have been made towards this goal by using yeast as an expression system for GPI‐anchored proteins, from which the GPI anchor is proteolytically released and purified.^271^ GPI‐anchored PrP was also found to quantitatively bind to DOPC vesicles. This emphasizes the contribution of GPI anchors in the membrane association of PrP.^257^ Noteworthy, the group of Silva et al is also working on intein‐based semisynthesis schemes to obtain homogeneous GPI‐anchored proteins, including PrP, using synthetic GPI anchors.^272^, ^273^, ^274^

A major limitation of obtaining semisynthetic PrP variants by EPL lies in the series of denaturation and renaturation steps required to obtain functional PrP‐intein fusion constructs due to expression into inclusion bodies in *E. coli*. The subsequent folding steps required for PrP^Palm^ and GPI‐anchored PrP also limits the overall yield of EPL reactions. Deposition in inclusion bodies in *E. coli* is probably due to misprocessing of newly generated PrP and the overproduction that impedes proper folding, including the formation of the structurally important disulfide bridge.^60^, ^275^ Hence, to improve the semisynthetic access to posttranslationally modified PrP Chu and Becker^261^ developed a strategy for soluble expression of PrP‐intein constructs in *E coli*. Ultimately, the overexpression of a PrP‐intein construct *N*‐terminally fused to the ATPase domain of heat shock protein (Hsp) 70 DnaK chaperone gave high quantities of soluble PrP. This approach offers an alternative way to produce PrP‐thioester for subsequent EPL reactions but also requires an additional step for removing the *N*‐terminal ATPase domain by using sortase A.

With robust semisynthetic strategies established, the critical membrane attachment of PrP was studied by Chu et al^241^ using three PrP variants, including full‐length FL_PrP (aa 23‐231), central hydrophobic region deleted ΔCR_PrP (aa 23‐231 with ∆105‐125) and *N*‐terminally truncated T_PrP (aa 90‐231), all equipped with a *C*‐terminal membrane anchor. Interactions of the lipidated PrP constructs with phospholipid membranes demonstrated binding modes distinct from the nonmodified PrPs and impacts on the biochemical and conformational properties of PrP. Whereas nonmodified PrPs showed a conversion into β‐sheet–enriched structures upon interaction with anionic POPG vesicles, lipidated ΔCR_PrP and T_PrP retained their α‐helical structure and lipidated FL_PrP partially converted into random coil. Evidence indicating pore formation of lipidated ΔCR_PrP was found in fluorescence‐based assays and supported by patch clamp electrophysiological measurements of cells transfected with lipidated ΔCR_PrP. ΔCR_PrP was previously found to be neurotoxic in vivo. Yet, expressed in cultured cells, it is identically localized as wild‐type PrP. Thus, altered binding interactions had been suggested to cause the deleterious signaling pathways.^276^, ^277^ Based on these results, critical roles for both *C*‐terminal membrane attachment and the *N*‐terminal domain of PrP have been suggested.

PTMs in PrP comprise not only a *C*‐terminal GPI anchor but also *N*‐glycosylation of two asparagine residues^278^ at positions 181 and 197. Different prion strains and prion‐related diseases (TSEs) possess distinct glycosylation patterns of PrP. Studying the influence of these PTMs in prion pathogenesis has not been forthcoming mainly due to the confusing complexity and heterogeneity of these glycans.^62^, ^279^ Shi et al^280^ reported a strategy based on linking three segments of murine PrP, in which a recombinant PrP segment (aa 90‐177S) was ligated with two synthetic peptide segments (aa 178‐212 and aa 213‐230). This strategy was aimed at introduction sugars into PrP but did not fully succeed. Shortly after that, Araman et al^255^ demonstrated a semisynthetic approach to generate PrP variants modified with monodisperse PEG chains as mimics of *N*‐glycans that are similar in size and molar mass (Figure 5C). A new EPL strategy was established to achieve this, in which expressed PrP (aa 23‐177) with a *C*‐terminal thioester is used in EPL reactions with PEGylated, synthetic peptides (aa 179‐231). Selective desulfurization of the β‐mercapto‐aspartate at the ligation site gives homogeneous PEGylated full‐length PrP constructs. Interestingly, in vitro aggregation was completely abrogated for all PEGylated PrP constructs under conditions at which wild‐type PrP aggregated. Furthermore, the addition of only 10% of PEGylated PrP completely blocked aggregation of wild‐type PrP. This has raised the question if large *N*‐glycans interfere with aggregation *in* *vivo*. Recently, Mishra et al^281^ introduced lactosyl and mannobiosyl glycans in huPrP (aa 90‐231) at positions 181 and 197 *via* Asn to Cys mutations. In agreement with our results, they found that glycosylated PrPs are less prone to spontaneous fibril nucleation. Such a strategy raises the question if added cysteine residues influence PrP structure by disulfide shuffeling and if this affects the modification reaction used by Mishra et al.^281^

A similar question can arise from the cysteine residues introduced during the EPL reactions described above as in our previous approach depicted in Figure 5A. The introduced ligation site cysteine at the *C*‐terminus of T_PrP (aa 90‐231) was left undesulfurized, which could potentially be problematic for the folding of PrP. To finally prove that such an additional *C*‐terminal cysteine residue does not influence PrP folding, we employed a strategy recently introduced by Matveenko et al,^256^ in which the two native cysteines in recombinant PrP (aa 23‐231) are protected by a phenacyl (PAc) protecting group. This protection allowed selective desulfurization of the introduced cysteine following EPL (Figure 5D). Comparing PrP variants containing a cysteine at the ligation site and an alanine (by CD) proved that the introduced cysteine did not disturb the folding to native PrP.

## 7 CONCLUSION AND OUTLOOK

Based on the continuous progress in protein (semi)synthesis, access to homogeneous, posttranslationally modified PrP variants was facilitated over the past decade and a set of differently modified variants could be characterized with respect to their biophysical and conformational properties, including their interaction with membranes. Semisynthetic PrP variants have the potential to shed light on the crucial steps in PrP conversion, transmission, and pathogenicity, eg., by allowing for direct observation of the protein at the cellular membrane. Understanding these key features in prion diseases can further serve as paradigm for other neurodegenerative diseases.
